# Supplementary material for: Self-attention enabled deep learning of dihydrouridine (D) modification on mRNAs unveiled a distinct sequence signature from tRNAs
Source: Mol Ther Nucleic Acids. 2023 Jan 27;31:411–20. doi: 10.1016/j.omtn.2023.01.014 (PMC9945750; doi:10.1016/j.omtn.2023.01.014)
Supplement: Document S1. Figures S1–S4 and Tables S1–S5 [file mmc1.pdf]

OMTN, Volume *31*

Supplemental information

Self-attention enabled deep learning of dihydrouridine (D) modification on mRNAs unveiled a distinct sequence signature from tRNAs

Yue Wang, Xuan Wang, Xiaodong Cui, Jia Meng, and Rong Rong

SUPPLEMENTAL TABLES

Table S1. Model performance of different deep learning methods for predicting dihydrouridine on mRNA.

**Modes**	**Methods**	**Sn** (%)	**Sp** (%)	**ACC** (%)	**F1**	**MCC**	**AUROC**
Training	Transformer	69.67	72.25	70.83	0.6986	0.3908	0.7362
	Local self-attention	80.55	77.78	79.17	0.7946	0.5895	0.8194
	CNN of one layer	90.74	85.19	87.96	0.8831	0.7608	0.8796
	CNN of two layers	90.02	86.67	88.33	0.8852	0.7674	0.8833
	ResNet	70.02	75.56	72.78	0.7158	0.4655	0.7873
	DPred	**92.59**	**87.78**	**90.27**	**0.8947**	**0.7826**	**0.9116**
Testing	Transformer	77.78	66.67	72.22	0.7368	0.4208	0.7654
	Local self-attention	83.33	72.22	77.78	0.7895	0.5590	0.8204
	CNN of one layer	88.89	77.78	86.11	0.8718	0.7324	0.8890
	CNN of two layers	94.44	83.33	88.89	0.8947	0.7826	**0.9035**
	ResNet	77.78	72.22	75.00	0.7568	0.5205	0.7870
	DPred	**94.44**	**88.89**	**91.66**	**0.9189**	**0.8062**	0.9027

Notes: DPred was compared with five other deep learning approaches, including local self-attention, CNNs, ResNet (residual connection + CNN) and Transformer (multi-heads self-attention). The results indicated that convolutional neural networks play crucial roles in site prediction. DPred, a combination of CNNs and local self-attention outperformed other methods that only used either one of them individually, and achieved the best performance in our study.

Table S2. DPred model performance on dihydrouridine tRNA datasets in different species.

Species	Sn (%)	Sp (%)	ACC (%)	F1 Scores	MCC	AUROC
S.cerevisiae	94.87	93.59	94.23	0.9427	0.8847	0.9675
H.sapiens	86.36	95.45	92.86	0.9315	0.8603	0.9731
E.coli	97.14	91.43	94.29	0.9444	0.8872	0.9857
M.musculus	92.43	86.77	91.67	0.9289	0.8346	0.9290

Table S3. DPred model performance under mature RNA / full transcript datasets.

Datasets	Sn (%)	Sp (%)	ACC (%)	F1 Scores	MCC	AUROC
Full transcript (101nt)	63.15	84.21	73.68	0.7059	0.4845	0.8333
mRNA (101 nt)	83.33	77.78	80.56	0.8108	0.6121	0.8642
Full transcript (41 nt)	78.94	89.47	84.21	0.8333	0.6880	0.8823
mRNA (41 nt)	94.44	88.89	91.66	0.9189	0.8062	0.9027

Table S4. EIIP value for each nucleotide.

Nucleotide	EIIP
A	0.1260
U	0.1335
G	0.0806
C	0.1340

Table S5. The proposed DPred architecture.

**Layers**	**Output size**
Input	(-, 41, 4)
Self_attention	(-, 41, 4)
Conv	(-, 41, 4, 100)
Max_pooling	(-, 20, 2, 100)
Flatten	(-, 4000)
Dropout	(-, 4000)
Dense	(-, 100)
Output	(-, 1)

SUPPLEMENTAL FIGURES

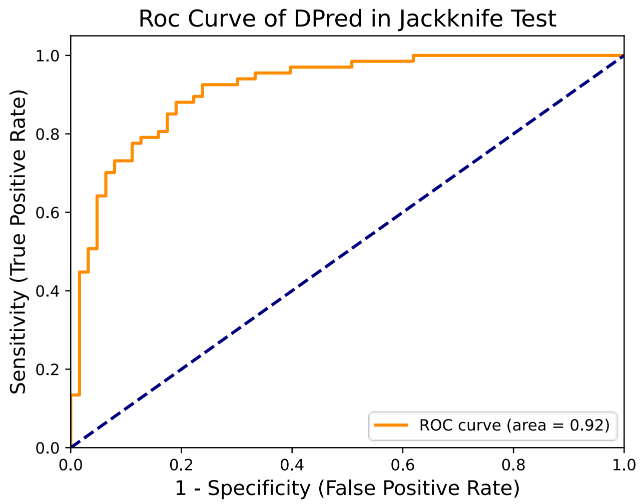

Figure S1. ROC curve on training data under jackknife cross-validation. The area under the receiver operating characteristic curve is 0.92.

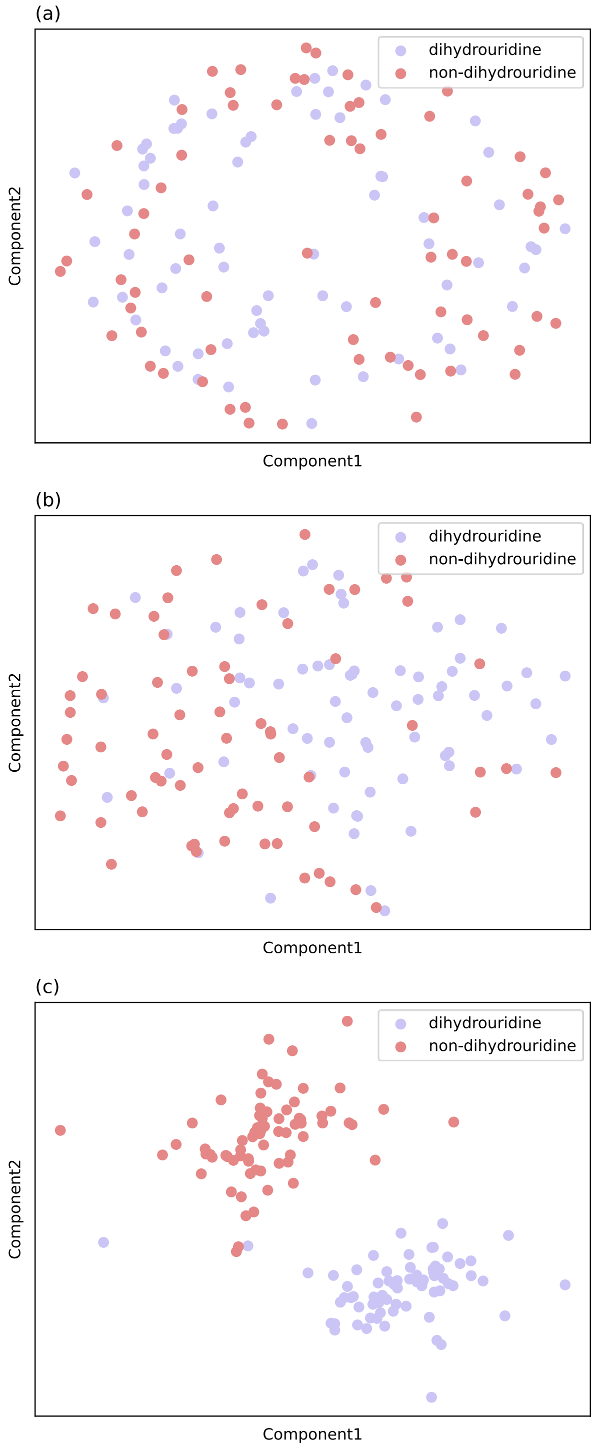

Figure S2. t-SNE plots of different layers in DPred. Feature visualization of (a) original input sequences, (b) flatten layer and (c) the last dense layer from the well-trained model.

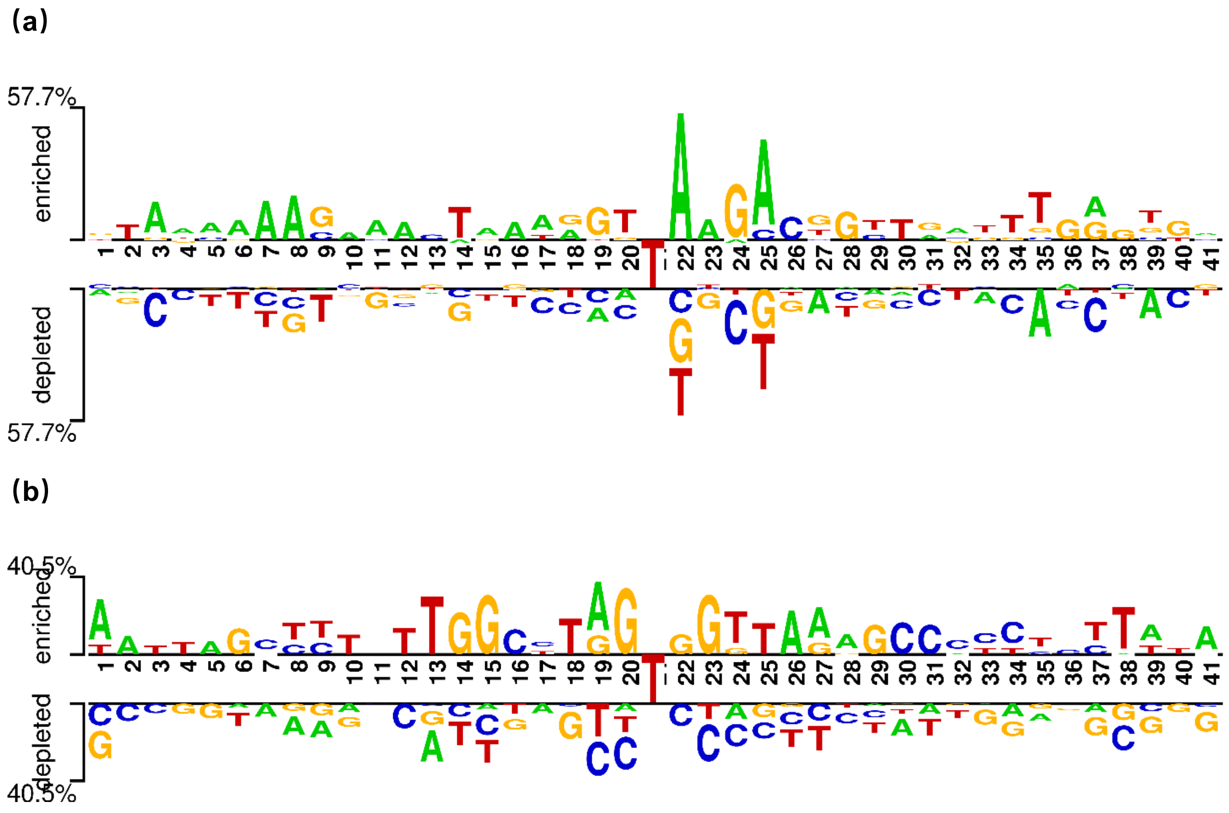

Figure S3. Two Sample Logo of the differences between positive and negative sequences. Demonstration of nucleotide composition preferences between dihydrouridine and non-dihydrouridine for mRNA (a) and tRNA (b) datasets.

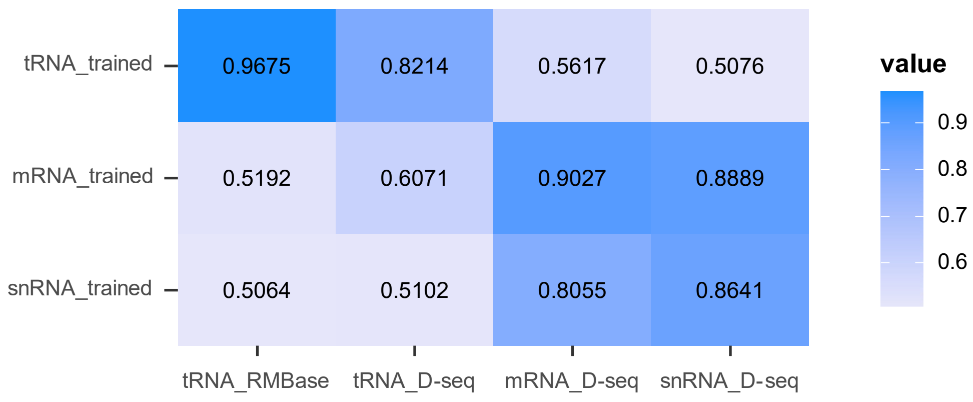

Figure S4. Heat map shows cross RNA types prediction accuracies. We trained DPred on tRNA, mRNA and snRNA training datasets independently, and made prediction on testing datasets of their own and other RNA types.
