# Supplementary material for: Self-attention enabled deep learning of dihydrouridine (D) modification on mRNAs unveiled a distinct sequence signature from tRNAs
Source: Mol Ther Nucleic Acids. 2023 Jan 27;31:411–20. doi: 10.1016/j.omtn.2023.01.014 (PMC9945750; doi:10.1016/j.omtn.2023.01.014)
Supplement: Document S2. Article plus supplemental information [file mmc2.pdf]

Molecular Therapy
Nucleic Acids
Original Article

Self-attention enabled deep learning of dihydrouridine (D) modification on mRNAs unveiled a distinct sequence signature from tRNAs

Yue Wang,[1,4] Xuan Wang,[2] Xiaodong Cui,[6] Jia Meng,[2,3,5] and Rong Rong[2]

[1]Department of Mathematical Sciences, Xi'an Jiaotong-Liverpool University, Suzhou, Jiangsu 215123, China; [2]Department of Biological Sciences, Xi'an Jiaotong-Liverpool University, Suzhou, Jiangsu 215123, China; [3]AI University Research Centre, Xi'an Jiaotong-Liverpool University, Suzhou, Jiangsu 215123, China; [4]Department of Computer Science, University of Liverpool, L69 7ZB Liverpool, UK; [5]Institute of Systems, Molecular and Integrative Biology, University of Liverpool, L69 7ZB Liverpool, UK; [6]School of Marine Science and Technology, Northwestern Polytechnical University, Xi'an, Shaanxi 710072, China

Dihydrouridine (D) is a modified pyrimidine nucleotide universally found in viral, prokaryotic, and eukaryotic species. It serves as a metabolic modulator for various pathological conditions, and its elevated levels in tumors are associated with a series of cancers. Precise identification of D sites on RNA is vital for understanding its biological function. A number of computational approaches have been developed for predicting D sites on tRNAs; however, none have considered mRNAs. We present here DPred, the first computational tool for predicting D on mRNAs in yeast from the primary RNA sequences. Built on a local self-attention layer and a convolutional neural network (CNN) layer, the proposed deep learning model outperformed classic machine learning approaches (random forest, support vector machines, etc.) and achieved reasonable accuracy and reliability with areas under the curve of 0.9166 and 0.9027 in jackknife cross-validation and on an independent testing dataset, respectively. Importantly, we showed that distinct sequence signatures are associated with the D sites on mRNAs and tRNAs, implying potentially different formation mechanisms and putative divergent functionality of this modification on the two types of RNA. DPred is available as a user-friendly Web server.

INTRODUCTION

Recent development of high-resolution transcriptome mapping has enabled the transcriptome-wide profiling of post-transcriptional RNA modification sites.[1] They were reported on almost all kinds of RNA, including mRNA, rRNA, tRNA, and small nuclear RNA (snRNA).[2,3] Over 170 post-transcriptional modifications have been identified and found to play important roles in various biological processes; e.g., fine-tuning RNA structures and functions, regulation of gene expression and protein synthesis, response to environmental exposures, cell differentiation, and mechanistic toxicology.[4–10] Post-transcriptional RNA modification can also have implications in human health and medical science.[11] To date, over 100 RNA modification enzyme mutations have been found to have an association with human diseases.[12]

Dihydrouridine (D) is a modified nucleotide universally found in viral, prokaryotic, and eukaryotic species. Recent studies showed that D is the second most prevalent modification in tRNA.[13,14] Catalyzed by D synthases (Dus), D is formed by the hydrogenation of uridine (U) whose C5–C6 bond is reduced, and is found within the eponym D loop of tRNAs.[15,16] With its unique structure, D can influence the RNA backbone and a series of RNA-involved processes. The modification can serve as a metabolic modulator for various pathological conditions, and its elevated levels in tumors are associated with a series of cancers in the lung,[17] liver,[18] kidney,[19] prostate,[20] and oral squamous cells.[21] It is a non-trivial task to accurately identify D in RNA and understand its fundamental biological function in all species.

With the development of new high-resolution mapping methods, more and more D sites have been identified on tRNAs in multiple species, including *Saccharomyces cerevisiae*, *Schizosaccharomyces pombe*, *Homo sapiens*, *Mus musculus*, *Escherichia coli*, *Bacillus subtilis*, and *Mycoplasma capricolum*. Database Web servers such as MODOMICS[1] and RMBase[22] were developed and greatly facilitated the study of tRNA modifications, in which D sites are included. Furthermore, wet-lab experimental methods such as D sequencing (D-seq) and rhodamine sequencing (Rho-seq) established the presence of D on mRNAs in yeast and human, which further verified its physiological significance.[14,23,24] Both methods take advantage of D-specific chemistry and next-generation sequencing to determine the location of D across the transcriptome, resulting in high-confidence single-nucleotide-resolution data.

Considering the high cost and long experiment time of wet-lab-based epitranscriptome profiling approaches, computational methods are often preferred as an alternative avenue.[25] A number of computational methods have been developed for predicting epigenetic

Received 22 September 2022; accepted 23 January 2023;
https://doi.org/10.1016/j.omtn.2023.01.014.

Correspondence: Rong Rong, Department of Biological Sciences, Xi'an Jiaotong-Liverpool University, Suzhou, Jiangsu 215123, China.
E-mail: rong.rong@xjtlu.edu.cn

This is an open access article under the CC BY license (http://creativecommons.org/licenses/by/4.0/).

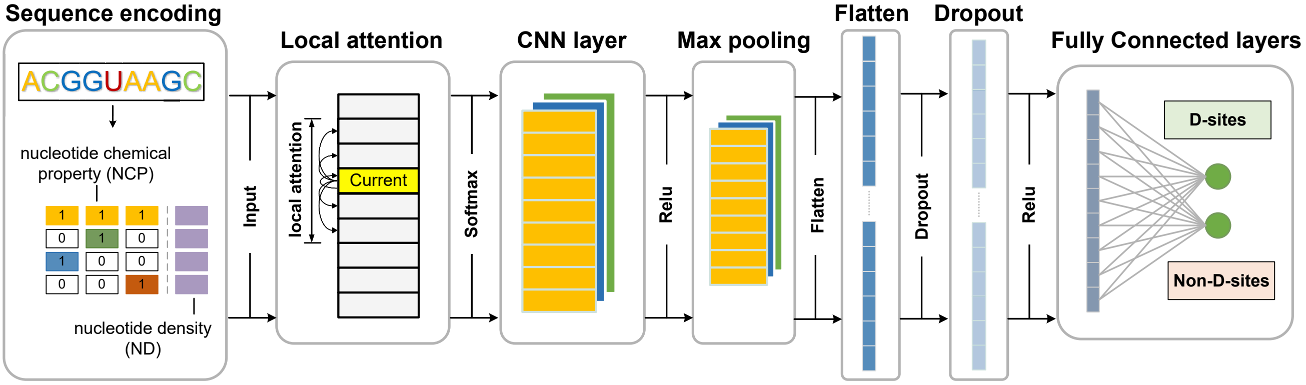

Figure 1. Model architecture

The model architecture mainly consists of local self-attention and convolutional mechanisms.

modifications of RNA.[26–37] Among them, iRNAD is the first approach for D-site prediction from multiple species, which used a support vector machine to distinguish D and non-D sites.[34] Later, iRNAD_XGBoost used XGBoost-selected multiple features to construct a model for D detection.[33] However, to the best of our knowledge, all existing D-site prediction tools[32–35] were trained on tRNAs, and it is not clear whether they can be applied to predict D sites on mRNAs. Although recent studies have unveiled the widely occurring nature and transcriptome-wide distribution of D (or the D epitranscriptome),[14] there are still no prediction tools constructed for mRNA D sites using mRNA D datasets.

Here we present DPred, the first computational method for predicting D modification on mRNAs in yeast from the primary RNA sequences. In DPred, the sequence data were encoded by the combination of nucleotide chemical property and nucleotide density, which outperformed three other encoding schemes. By taking advantages of local self-attention mechanisms and convolutional neural networks (CNNs), DPred can effectively distinguish D-containing sites from the non-modified by exploiting informative features directly from primary sequences. It achieved reasonable accuracy and reliability with areas under the curve (AUCs) of 0.9166 and 0.9027 in jackknife cross-validation and on independent testing dataset, respectively. A simplified graphic framework of DPred is illustrated in Figure 1. Additionally, we showed that the sequence contexts of D sites on mRNAs and tRNAs are substantially different, and the D-prediction tools trained by mRNA and tRNA data should be clearly distinguished.

To facilitate the access of our predictor, a user-friendly Web server has also been developed and made freely available at http://www.rnamd.org/dpred. It is expected that our predictor could be a useful tool for researchers interested in D modification.

RESULTS

We developed the first mRNA D predictor enabled by deep neural networks. We established our benchmark dataset based on the high-confidence single-nucleotide-resolution data reported by the D-seq technique.[23] Each unmodified site was randomly selected on the same transcript of each positive D site, making the positive-to-negative ratio 1:1. The dataset was randomly split into the training and testing part with a ratio of 8:2. Experiments were performed under jackknife cross-validation in the training phase.

Choosing a good encoding strategy is crucial for constructing a high-accurate predictor. Two nucleotide representation methods: one hot (OH), nucleotide chemical property (NCP), and two properties, namely nucleotide density (ND) and electron-ion interaction potential (EIIP), were employed in this study. Each nucleotide representation and each nucleotide property formed an encoding strategy. Thus, four encoding schemes, OH_ND, OH_EIIP, NCP_ND, and NCP_EIIP were compared in the following experiments and discussion. Details can be found in the "materials and methods" section.

The deep neural network model mainly consists of an additive local self-attention layer and a CNN layer (see Figure 1). The attention layer uses additive self-attention to calculate the alignment weights indicating how much attention it should give to each input state. The CNN layer exploits local sequence motifs for a given target through its local receptive fields. A max-pooling layer is followed to filter weak features and expand the receptive field. A dropout layer is implemented to avoid overfitting in model training. A fully connected layer eventually takes all the previous results and leads to a softmax function with a 0.5 threshold, which predicts whether the input sequence contains D sites or not. For more details, please see the "materials and methods" section.

Model performance evaluation and comparison with other methods

We evaluated proposed DPred and other competing classifiers on the benchmark dataset stated above with different encoding schemes. Results are summarized in Table 1. The competing methods include logistic regression (LR), random forest (RF), support vector machine (SVM), and XGBoost (XG). Model performance was evaluated by jackknife cross-validation and independent testing set. When using NCP-based encoding strategies, classifiers showed more reliable and

Table 1. Comparison of different methods and encoding methods (AUROC)

Modes	Methods	LR	RF	SVM	XG	DPred
NCP_ND	cross-validation	0.8686	0.8958	0.8449	0.8958	0.9166
	testing	0.8242	0.8981	0.8056	0.8827	0.9027
NCP_EIIP	cross-validation	0.8282	0.9275	0.8429	0.8750	0.9043
	testing	0.8194	0.8734	0.8163	0.8688	0.8889
OH_ND	cross-validation	0.8132	0.8645	0.7747	0.8560	0.8571
	testing	0.7778	0.8518	0.7944	0.8256	0.8395
OH_EIIP	cross-validation	0.8177	0.8428	0.7944	0.8583	0.8214
	testing	0.8009	0.8611	0.7880	0.8441	0.8056

robust results. The RF and XGBoost classifiers generally outperformed LR and SVM. In the case of OH-based encoding, the RF and XGBoost even achieved better accuracy than our DPred. In NCP_ND, DPred outperformed the competing classifiers and achieved the highest accuracy among all the tests. It obtained AUCs of 0.9166 and 0.9027 in jackknife cross-validation on the training dataset and on independent testing dataset, respectively. The receiver operating characteristic (ROC) curve is plotted in Figure S1. We also compared DPred with a series of deep learning neural networks. Results were summarized in Table S1. We found that our DPred, a combination of CNNs and local self-attention, outperformed other methods that only used either one of them individually, and it achieved the best performance in our study. Details of other methods can be viewed on GitHub.

To give an intuitive assessment of the classification ability, we drew t-SNE (t-distributed Stochastic Neighbor Embedding) plots generated by different layers in DPred, which visualized the distance between samples after dimensionality reduction, as shown in Figure S2. The positive samples (red dots) are gradually clustered in the upper-left area through leaning, whereas the negative samples are concentrated in the lower-right area. As shown in the t-SNE plots, our proposed method is capable of extracting features that can efficiently discriminate D sites from non-D sites.

Since the available high-quality datasets are limited, this study has not involved multi-species prediction of mRNA D sites. To further demonstrate the generalization of proposed method, we collected available tRNA D sites reported by RMbase[22] and MODOMICS[1] in different species, including *S. cerevisiae*, *H. sapiens*, *M. musculus*, and *E. coli*. Results showed that the DPred framework achieved good performance in all datasets, which are summarized in Table S2.

The performance of our predictor was evaluated on the full transcript mode and mRNA mode. The full transcript mode kept both exonic and intronic regions of the positive and negative sites, while, in the mRNA mode, only the exons were kept. The sequences utilized in both modes were based on the same D and non-modified sites. We eventually found mRNA-based prediction outperformed full transcript-based prediction on both modes of 41 nt and 101 nt lengths. Results are summarized in Table S3.

Prediction tools of D sites trained by mRNA and tRNA data should be clearly distinguished

To the best of our knowledge, all existing proposed D-site prediction tools were trained on tRNAs, while none considered mRNAs. It is of interest to explore whether a tRNA-trained model can be applied to predict D sites on mRNAs, and also the reverse of the case. We therefore performed prediction for D over mRNA (tRNA) sequences using the DPred approach trained by tRNA (mRNA) sequences. The D-containing tRNA sequences in yeast were collected from the database RMBase 2.0.[22] Interestingly, we found these two types of datasets basically cannot be used to predict each other (Table 2). This finding indicated that the D-prediction tools trained by mRNA and tRNA data should be clearly distinguished.

Analysis of nucleotide composition preference

Importantly, we revealed that the sequence contexts of D sites on mRNAs and tRNAs are substantially different, which explained why D-related mRNA and tRNA datasets cannot be used to train models that predict each other. We demonstrated the position-specific differences around D sites on mRNA, snRNA, and tRNA sequences respectively in Figure 2. On tRNA sequences, G bases are enriched around D sites, whereas, on mRNA sequences, this pattern is no longer the same, and A bases are found to be over-represented at positions 1 and 4. The plots were drawn by the tool ggseqlogo.[38] Furthermore, we conducted motif analysis between positive and negative sequences on tRNAs and mRNAs respectively by the graphical tool two-sample-logo,[39] and showed the differences in nucleotide distributions around D and non-D sites, suggesting it is feasible to design a computational model identifying the D only based on the primary sequences (Figure S3).

Based on the datasets we collected, we also conducted mixed prediction about D sites between snRNA/tRNA/mRNA sequences and drew a heatmap with the accuracies shown in Figure S4. The RMBase tRNA-trained model achieved good performance on both RMBase and D-seq tRNA datasets but failed to predict D sites on mRNA and snRNA. On the contrary, mRNA- and snRNA-trained models made reliable predictions on their own datasets but could not classify tRNA D sites well. This is consistent with the motif analysis in Figure 2, where the nucleotide compositions around the D sites on mRNA and snRNA sequences are considerably different from that on tRNAs.

Table 2. Performance evaluation results of D sites on tRNA (mRNA) predicted by mRNA (tRNA) data

Training data	Testing data	Sn (%)	Sp (%)	ACC (%)	F1 scores	MCC	AUROC
tRNA	mRNA	22.22	83.33	52.77	0.3200	0.0702	0.5617
mRNA	tRNA	15.38	84.62	51.92	0.2352	0.0494	0.5192
tRNA	tRNA	94.87	93.59	94.23	0.9427	0.8847	0.9675
mRNA	mRNA	94.44	88.88	91.66	0.9189	0.8062	0.9027

Web implementation

DPred takes FASTA sequences as input. Users can paste the sequences into the text box on the Web site or upload a FASTA file (Figure 3). The input sequence should be 41 nt long, and in the center should be a potential D site to be evaluated. The Web server will predict the D-site possibility using our well-trained DPred model. Results can be downloaded as an Excel table.

DISCUSSION

In this study, we developed the first computational tool for prediction of D sites on mRNAs in yeast. We explored different ways to encode RNA so as to capture the informative features within each sequence. The model framework was mainly built on the additive local self-attention and CNN architecture. Accuracies of 0.9166 and 0.9027 were achieved in cross-validation and an independent testing dataset, showing reasonable accuracy and reliability of our model.

The universal D modification is first discovered within the eponym D loop of tRNAs. It results from a reduction of the uridine C5–C6 bond by Dus enzymes. Its unique structure leads to a series of special biochemical properties, including the potential destabilization of the RNA structure and molecular flexibility.[14] Particularly, D affects the flexibility of tRNAs. It involves the formation of cloverleaf-related tRNA secondary structure and L-shaped tRNA tertiary structure by causing changes to the RNA backbone conformation.[40,41]

Recent studies show that the D distribution may spread to other types of RNA. Newly developed high-resolution mapping methods established the presence of D within mRNAs, which further verified its physiological significance.[14,23,24] D-seq reported that certain (not all) mRNA D sites occur in regions where secondary structure potential is evolutionarily conserved, suggesting biological function possibly analogous to D sites on tRNAs.[23] Another question is raised concerning how D on mRNA involves translation. An interesting finding is that a dihydrouridylated mRNA could be translationally repressed, where D may modulate the translational speed and ribosome stalling.[42] A conjecture to explore here is that a D on mRNA enables co-translational folding by regulating the speed of translation.[14] However, detailed biological functions and formation mechanisms of modified mRNAs still need to be investigated.

Interestingly, in our study, we found that the D sites on mRNAs and tRNAs have distinct sequence contexts. Although this may be due to technical bias embedded in data obtained from different resources, a more exciting hypothesis may be that the D sites on mRNAs and tRNAs may have different formation mechanisms and fulfill divergent biological functions, which remains to be tested in the future. The expression level of Dus enzymes and the D landscape will be the subject of further investigation of the roles dihydrouridylated mRNAs and tRNAs play in translation and other physiological activities.[14]

Since high-quality D epitranscriptome data are limited, multi-species prediction has not been conducted in this study. Other features, such as secondary structures, genome information, and RNA types, will need to be involved to further improve the robustness and generalization ability of the model in the future.

MATERIALS AND METHODS

Benchmark datasets

The benchmark dataset needs to include both positive and negative sequences. The sequences should contain a uridine in the middle that is a potential D modification to be evaluated; i.e., the sequence should be $2\gamma + 1$ long, where γ is the number of nucleotides from center to both sides. The D-containing mRNA sequences (positive datasets) were obtained from the recently published single-nucleotide-resolution D-seq data.[23] The D-containing tRNA sequences were collected from the database RMBase 2.0.[22] Unmodified sequences (negative datasets) were randomly selected on the same transcripts of the positive D sites. There are a total of 956 RNA samples, of which 780 are tRNA sequences and 176 are mRNA sequences. Both have a 1:1 positive-to-negative ratio. We randomly split the dataset into training and testing datasets with a ratio of 8:2. Experiments were performed under jackknife cross-validation in the training phase. Each dataset contains an equal number of positive and negative sequences.

Furthermore, if a potential D site is near the 5′ or 3′ end, a head-to-end principle is utilized when extracting corresponding sequences.[34] If the distance from a potential D site to the transcript head (end) is less than the window size γ, the missing part is filled by the transcript end (head) part nucleotides. This strategy ensures all sequences to be extracted have the same length and the D/uridine in the center.

Feature extraction strategy

In this study, four encoding schemes were employed, which were different combinations of nucleotide representations and properties.

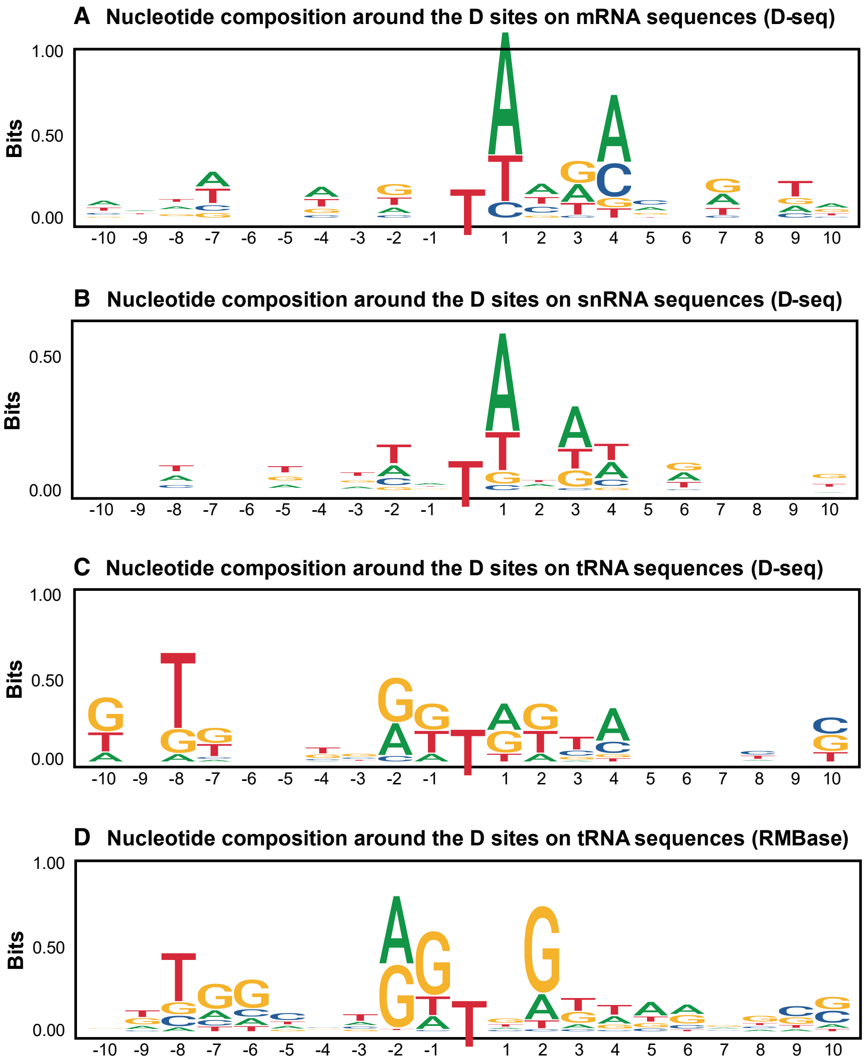

Figure 2. Demonstration of nucleotide composition preference

(A–C) The sequence contexts around D modification sites on mRNA, snRNA, and tRNA sequences. (A), (B), and (C) were derived from D-seq data.[23] (D) was based on RMBase data.[22] D sites on mRNAs and tRNAs have distinct sequence signatures, which are quite informative. On the contrary, the sequence signatures of D sites on tRNA from D-seq technology and RMBase are very similar, suggesting technical bias should not be the major cause of the previous discrepancy.

The nucleotide representation methods included OH and NCP. The nucleotide properties included ND and EIIP. Thus, we compared four encoding strategies, OH_ND, OH_EIIP, NCP_ND, and NCP_EIIP, to explore which combination of encoding strategies led to the best performance in the model training. Details of them are stated below.

OH is the most common feature representation method and maps each element into a vector. In this study, there are four types of nucleotide: A (adenine), C (cytosine), G (guanine), and U (uracil). Each nucleotide in sequences can be assigned to a vector of 4 ($A \rightarrow [1,0,0,0], C \rightarrow [0,1,0,0], G \rightarrow [0,0,1,0], U \rightarrow [0,0,0,1]$).

NCP is a nucleotide representation approach based on the chemical structures of RNA nucleotides. It was first proposed for site prediction in DNA sequences and now is also widely used in RNA sequences.[43–45] Four different types of nucleotide are classified into three groups: the number of ring structure (A and G have two rings, while C and U have one ring), the chemical functionality (A and C have an amino group, G and U have a keto group), and the hydrogen bond strength (hydrogen bonding between C and G is strong, that between A and U is weak). The i-th nucleotide in a sequence is denoted by $N_i = (x_i, y_i, z_i)$, where s_i is nucleotide type and x_i, y_i, z_i reflect these three groups respectively:

$$x_i = \begin{cases} 1 & \text{if } s_i \in (A, G) \\ 0 & \text{if } s_i \in (C, U) \end{cases} \qquad \text{(Equation 1)}$$

$$y_i = \begin{cases} 1 & \text{if } s_i \in (A, C) \\ 0 & \text{if } s_i \in (G, U) \end{cases} \qquad \text{(Equation 2)}$$

$$z_i = \begin{cases} 1 & \text{if } s_i \in (A, U) \\ 0 & \text{if } s_i \in (C, G) \end{cases} \qquad \text{(Equation 3)}$$

ND indicates the distribution and cumulative frequency information of nucleotides at each position.[46] The density of the i-th nucleotide is calculated as the number of nucleotides that have the same types as before the $i+1$-th position, divided by i:

$$d_i = \frac{\sum_{k=1}^{i} f(s_k)}{i} \qquad \text{(Equation 4)}$$

where

$$f(s_k) = \begin{cases} 1 & \text{if } s_k = s_i \\ 0 & \text{otherwise} \end{cases} \qquad \text{(Equation 5)}$$

The EIIP value of the nucleotides was first proposed in 2006,[47] indicating the electron-ion interaction potential of an mRNA nucleotide, and later was utilized by m^5 C prediction[48] and other bioinformatics research areas.[49,50] The EIIP values for different nucleotides are given in Table S4.

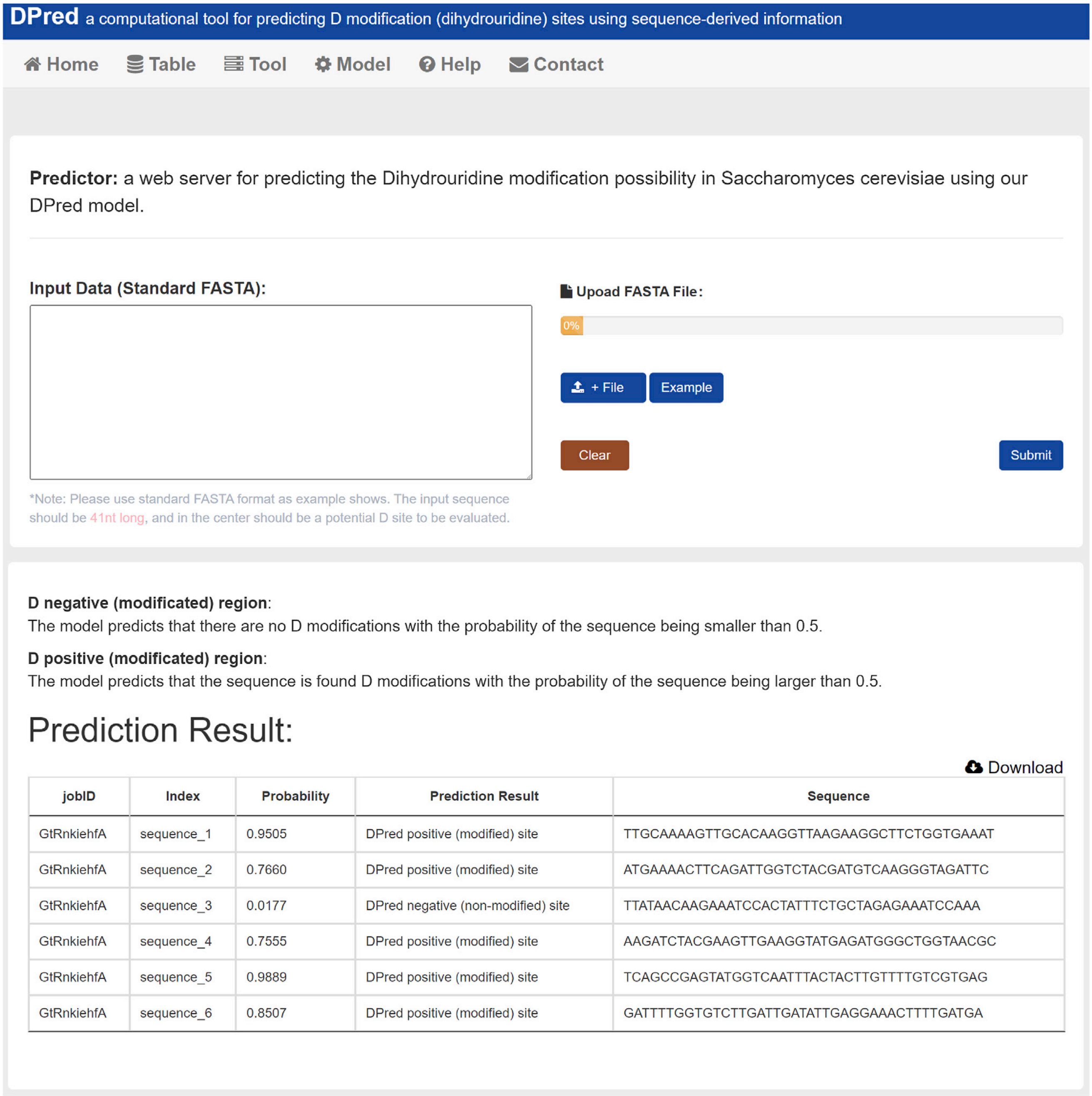

Figure 3. Screenshot of the DPred Web server

Users can upload the query sequences. Results will then be presented after a while.

Each nucleotide in sequences was encoded into a discrete vector consisting of a nucleotide representation plus a nucleotide property. For example, in OH_EIIP, A, C, G, and U were encoded as vectors [1, 0, 0, 0, $EIIP_i$], [0, 1, 0, 0, $EIIP_i$], [0, 0, 1, 0, $EIIP_i$], and [0, 0, 0, 1, $EIIP_i$]. Each sequence was encoded as a $2\gamma + 1 \times 5$ matrix. In NCP_ND, A, C, G, and U were encoded as vectors [1, 1, 1, d_i], [0, 1, 0, d_i], [0, 0, 1, d_i], and [0, 0, 1, d_i]. Each sequence was encoded as a $2\gamma + 1 \times 4$ matrix. In the following discussion, we explore which combination of encoding strategies leads to the best performance in the model training.

Performance evaluation metrics

The model performance was evaluated by various statistical metrics, including sensitivity (Sn, also termed recall), specificity (Sp), accuracy (ACC), F1 scores, and Mathew's correlation coefficient (MCC):

$$S_n = Re = \frac{TP}{TP + FN} \quad \text{(Equation 6)}$$

$$S_p = \frac{TN}{TN + FP} \quad \text{(Equation 7)}$$

$$ACC = \frac{TP + TN}{TP + TN + FP + FN} \quad \text{(Equation 8)}$$

$$Pre = \frac{TP}{TP + FP} \quad \text{(Equation 9)}$$

$$F_1 = 2 * \frac{Pre * Re}{Pre + Re} \quad \text{(Equation 10)}$$

$$MCC = \frac{TP \times TN - FP \times FN}{\sqrt{(TP + FP) \times (TP + FN) \times (TN + FP) \times (TN + FN)}} \quad \text{(Equation 11)}$$

TP, TN, FP, and FN represent the number of true-positive, true-negative, false-positive, and false-negative, respectively. Re and Pre indicate recall and precision. The ROC curve and the area under the ROC (AUROC) were also included to quantitatively evaluate the proposed method.

Sequence length and feature extraction optimization

It is crucial to select a suitable input sequence length for the prediction model. If the length is too short, input sequences cannot provide enough information. If the sequence length is too long, redundant information could be introduced, increasing the difficulty for the model to extract crucial features. The encoding schemes can influence model performance as well and also need to be selected. Thus, we evaluated DPred under different sequence lengths and encoding schemes.

The range of sequence length to be evaluated is from 11 to 81 nt. Figure 4 shows the performance evaluation results based on different sequence sizes and four encoding schemes. We found the two NCP-based encoding methods generally outperformed the OH-based methods. In NCP_EIIP, the model achieved the best performance with an AUC of 0.8992 when the sequence size was 31 nt. In NCP_ND, an AUC of 0.8889 was obtained when the length was 31 nt, and a 0.9027 AUC was attained at 41 nt. We chose 41 nt as the sequence length and NCP_ND as the final encoding strategy for the predictor.

The ROC curves for the independent testing dataset are plotted in Figure 5. Model performance was evaluated based on 41-nt sequence length for four encoding methods. NCP_ND generally yielded the best prediction among the four encoding schemes.

Model architecture

The network framework is as follows: the first local attention[51] layer uses additive attention and assigns attention scores to each state in the sequence; a convolutional layer exploits meaningful features in its local receptive fields by using weight-sharing strategy; a max-pooling layer removes unimportant features and enlarges the receptive field; all processed features are then flattened and fed into a dropout layer to avoid overfitting in model training and to increase the generalization of the network on unknown sequences. A fully connected layer eventually takes all the previous results and leads to a softmax function, which predicts whether the input sequence contains D sites or not with a 0.5 threshold. The rectified linear unit (ReLU) is used as an activation function throughout the framework except for the first local attention layer and the last layer, which utilize the softmax function.

L2 regularization was adopted in the dense layer. During the L2 regularization, the loss function of the neural network is extended by a regularization term, which is the L2 norm of the weight matrices; i.e., the sum over all squared weight values. The regularization term is weighted by a regularization rate being set to 0.01. Performing L2 regularization makes the weights of the neural network small (toward zero but not exactly zero). Smaller weights reduce the impact of the hidden neurons and lower the complexity of the model during training.

Attention-based methods have gained popularity in neural networks since the late 2010s. The attention structure highlights the importance of key points relative to their neighborhood regions and makes the model learn what to attend to from the given sequence.[52] Self-attention was one of the attention mechanisms that attends to states within the sequence[53]; i.e., it only focuses on how one state attends to other states within a given sequence. Research has found that self-attention layers operate similarly to CNN layers[54] but are more flexible with a learnable receptive field. This mechanism has been widely used in image analysis and NLP (Natural Language Processing) tasks, sometimes combing with or replacing CNNs, RNNs (Recurrent Neural Networks), and LSTMs (Long Short-Term Memory Networks).

Luong et al.[51] first distinguished between global attention[52] versus local attention. Global attention considers all the states, while the local attention only considers a subset of states when calculating attention weights. The latter combines the strength of both hard and soft attention and is differentiable, hence easier to implement and train.

The attention layer used in this model is the additive local self-attention. In this model, let x_i and x_j denote the i-th and the j-th element in the sequence respectively. It should first calculate the alignment (attention) weights $a_{i,j}$, which is derived by aligning the state i with the state j:

$$g_{i,j} = \tanh\left(x_i W_q{}^T + x_j W_k{}^T + b_g\right) \quad \text{(Equation 12)}$$

$$e_{i,j} = g_{i,j} W_a{}^T + b_a \quad \text{(Equation 13)}$$

$$a_{i,j} = \mathrm{softmax}\left(e_{i,j}\right) = \frac{\exp\left(e_{i,j}\right)}{\sum_j \exp\left(e_{i,j}\right)} \quad \text{(Equation 14)}$$

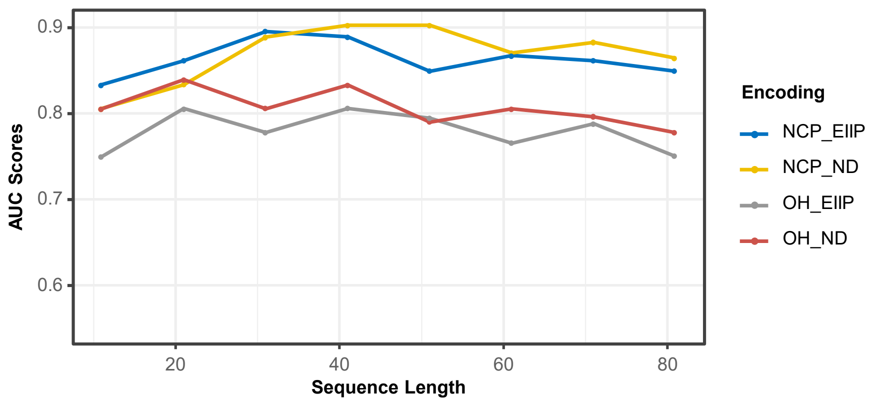

Figure 4. Sequence length optimization by AUROC
Model performance was evaluated with different sequence lengths and encoding schemes by comparing AUROC values. 41nt sequence length and NCP_ND encoding scheme were chosen for the predictor.

The $e_{i,j}$ is alignment score function. Here, it used an additive structure, which is why we called this layer an additive local self-attention. Besides this additive structure, the alignment score function can also take dot product, scaled dot product, and cosine similarity operation. Furthermore, the local attention layer only focuses on a subset of context, which means, for each state i, we should not calculate the alignment of every state in the sequence with it, but only part of them with it. We used monotonic alignment here,[51] just setting the aligned position p_i to be i; i.e., the i-th state is aligned with a window of states around itself. For example, if the window size is 5, each state in the sequence is compared with two states in front of it, with two states behind it, and with itself, respectively. Next, the alignment weights were obtained by feeding alignment scores through a softmax function. The weight indicates how much attention it should give to each input state.

The states in the input layer were weighted to give a new vector that is used by the subsequent layer. The new vector is this attention-focused state representation l_i, derived by the weighted average over the input sequence, which should have the same size with the input layer.

$$l_i = \sum_j a_{i,j} x_j \quad \text{(Equation 15)}$$

Selected hyper-parameters are presented in Table 3. The layers of the model and each output shape are summarized in Table S5. The loss function in this model computed a categorical cross entropy loss:

$$\text{Loss} = -\sum_{s=1}^{N} y_s \cdot \log \hat{y}_s \quad \text{(Equation 16)}$$

where y_s is the true label of the s-th sample, $\hat{y}_s$ is the prediction, and N is the number of values in the model output (2 in this study). The Adam optimizer,[55] which combines the strength of momentum and adaptive learning rate method, was used for training the network with a small initial learning rate of 0.0001. The epoch was set to be 100.

DATA AND CODE AVAILABILITY

The DPred framework was implemented using Tensorflow 2.10.0, and the codes can be freely accessed at https://github.com/yue-wang-biomath/DPred. The corresponding Web server can be assessed from http://www.rnamd.org/dpred.

SUPPLEMENTAL INFORMATION

Supplemental information can be found online at https://doi.org/10.1016/j.omtn.2023.01.014.

ACKNOWLEDGMENTS

This project was supported by the National Science Foundation for Young Scientists of China (grant no. 62003273) and Natural Science Basic Research Program of Shaanxi (program no. 2020JQ-217).

AUTHOR CONTRIBUTIONS

J.M. initialized the project. Y.W. and J.M. conceived the ideas. Y.W. collected and processed all of the data. Y.W. designed and implemented the model. X.W. built the Web server. Y.W. performed the analysis and compared it with competing methods. Y.W. drafted

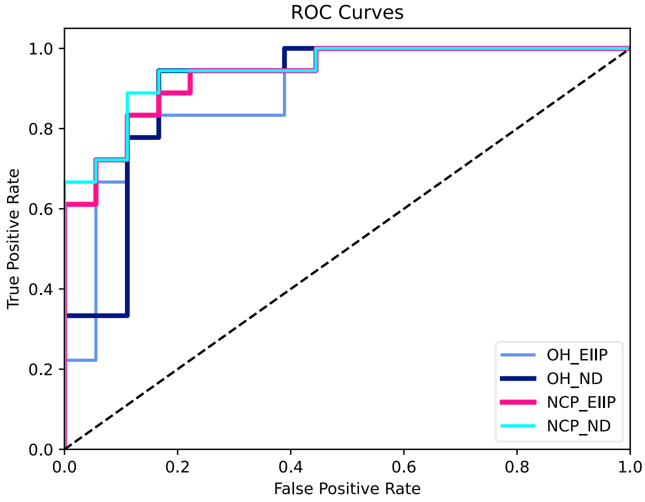

Figure 5. ROC curves of DPred performance
The curves were drawn based on the model performance with 41nt sequence length and four encoding methods in the testing dataset.

Table 3. Selected hyper-parameters for the DPred model

Hyper-parameters	Selected values
Attention type	additive
Attention hidden size	32
Attention width	2
Number of filters	100
CNN stride size	2
Kernel size	2
Padding	same
Max-pooling stride-size	1
Max-pooling pool-size	2
Dropout ratio	0.1
Regularization rate	0.01
Dense size	100
Loss function	categorical_crossentropy
Optimizer	Adam
Learning rate	0.0001

the manuscript. All authors read, critically revised, and approved the final manuscript.

DECLARATION OF INTERESTS

The authors declare no competing interests.

REFERENCES

1. Boccaletto, P., Stefaniak, F., Ray, A., Cappannini, A., Mukherjee, S., Purta, E., Kurkowska, M., Shirvanizadeh, N., Destefanis, E., Groza, P., et al. (2022). MODOMICS: a database of RNA modification pathways. 2021 update. Nucleic Acids Res. *50*, D231–D235.
2. Helm, M., and Alfonzo, J.D. (2014). Posttranscriptional RNA modifications: playing metabolic games in a cell's chemical legoland. Chem. Biol. *21*, 174–185.
3. Ma, J., Song, B., Wei, Z., Huang, D., Zhang, Y., Su, J., de Magalhães, J.P., Rigden, D.J., Meng, J., and Chen, K. (2022). m5C-Atlas: a comprehensive database for decoding and annotating the 5-methylcytosine (m5C) epitranscriptome. Nucleic Acids Res. *50*, D196–D203.
4. Batista, P.J., Molinie, B., Wang, J., Qu, K., Zhang, J., Li, L., Bouley, D.M., Lujan, E., Haddad, B., Daneshvar, K., et al. (2014). m6A RNA modification controls cell fate transition in mammalian embryonic stem cells. Cell Stem Cell *15*, 707–719.
5. Delaunay, S., and Frye, M. (2019). RNA modifications regulating cell fate in cancer. Nat. Cell Biol. *21*, 552–559.
6. Yang, C. (2020). ToxPoint: dissecting functional RNA modifications in responses to environmental exposure—mechanistic toxicology research enters a new era. Toxicol. Sci. *174*, 1–2.
7. Pendleton, K.E., Chen, B., Liu, K., Hunter, O.V., Xie, Y., Tu, B.P., and Conrad, N.K. (2017). The U6 snRNA m6A methyltransferase METTL16 regulates SAM synthetase intron retention. Cell *169*, 824–835.e14.
8. Liu, N., Dai, Q., Zheng, G., He, C., Parisien, M., and Pan, T. (2015). N6-methyladenosine-dependent RNA structural switches regulate RNA–protein interactions. Nature *518*, 560–564.
9. Geula, S., Moshitch-Moshkovitz, S., Dominissini, D., Mansour, A.A., Kol, N., Salmon-Divon, M., Hershkovitz, V., Peer, E., Mor, N., Manor, Y.S., et al. (2015). Stem cells. m6A mRNA methylation facilitates resolution of naïve pluripotency toward differentiation. Science *347*, 1002–1006.
10. Song, B., Huang, D., Zhang, Y., Wei, Z., Su, J., Pedro de Magalhães, J., Rigden, D.J., Meng, J., and Chen, K. (2022). m6A-TSHub: unveiling the context-specific m6A methylation and m6A-affecting mutations in 23 human tissues. Dev. Reprod. Biol. https://doi.org/10.1016/j.gpb.2022.09.001.
11. Song, B., Wang, X., Liang, Z., Ma, J., Huang, D., Wang, Y., de Magalhães, J.P., Rigden, D.J., Meng, J., Liu, G., et al. (2022). RMDisease V2.0: an updated database of genetic variants that affect RNA modifications with disease and trait implication. Nucleic Acids Res. gkac750.
12. Jonkhout, N., Tran, J., Smith, M.A., Schonrock, N., Mattick, J.S., and Novoa, E.M. (2017). The RNA modification landscape in human disease. RNA *23*, 1754–1769.
13. Machnicka, M.A., Olchowik, A., Grosjean, H., and Bujnicki, J.M. (2014). Distribution and frequencies of post-transcriptional modifications in tRNAs. RNA Biol. *11*, 1619–1629.
14. Finet, O., Yague-Sanz, C., Marchand, F., and Hermand, D. (2022). The dihydrouridine landscape from tRNA to mRNA: a perspective on synthesis, structural impact and function. RNA Biol. *19*, 735–750.
15. Byrne, R.T., Jenkins, H.T., Peters, D.T., Whelan, F., Stowell, J., Aziz, N., Kasatsky, P., Rodnina, M.V., Koonin, E.V., Konevega, A.L., and Antson, A.A. (2015). Major reorientation of tRNA substrates defines specificity of dihydrouridine synthases. Proc. Natl. Acad. Sci. USA *112*, 6033–6037.
16. Yu, F., Tanaka, Y., Yamashita, K., Suzuki, T., Nakamura, A., Hirano, N., Suzuki, T., Yao, M., and Tanaka, I. (2011). Molecular basis of dihydrouridine formation on tRNA. Proc. Natl. Acad. Sci. USA *108*, 19593–19598.
17. Kato, T., Daigo, Y., Hayama, S., Ishikawa, N., Yamabuki, T., Ito, T., Miyamoto, M., Kondo, S., and Nakamura, Y. (2005). A novel human tRNA-dihydrouridine synthase involved in pulmonary carcinogenesis. Cancer Res. *65*, 5638–5646.
18. Kuchino, Y., and Borek, E. (1978). Tumour-specific phenylalanine tRNA contains two supernumerary methylated bases. Nature *271*, 126–129.
19. The Cancer Genome Atlas Research Network (2013). Comprehensive molecular characterization of clear cell renal cell carcinoma. Nature *499*, 43–49.
20. Huang, J., Mondul, A.M., Weinstein, S.J., Derkach, A., Moore, S.C., Sampson, J.N., and Albanes, D. (2019). Prospective serum metabolomic profiling of lethal prostate cancer. Int. J. Cancer *145*, 3231–3243.
21. Sridharan, G., Ramani, P., and Patankar, S. (2017). Serum metabolomics in oral leukoplakia and oral squamous cell carcinoma. J. Cancer Res. Ther. *13*, 556–561.
22. Xuan, J.-J., Sun, W.-J., Lin, P.-H., Zhou, K.-R., Liu, S., Zheng, L.-L., Qu, L.-H., and Yang, J.-H. (2018). RMBase v2.0: deciphering the map of RNA modifications from epitranscriptome sequencing data. Nucleic Acids Res. *46*, D327–D334.
23. Draycott, A.S., Schaening-Burgos, C., Rojas-Duran, M.F., Wilson, L., Schärfen, L., Neugebauer, K.M., Nachtergaele, S., and Gilbert, W.V. (2022). Transcriptome-wide mapping reveals a diverse dihydrouridine landscape including mRNA. PLoS Biol. *20*, e3001622.
24. Finet, O., Yague-Sanz, C., and Hermand, D. (2022). Epitranscriptomic mapping of RNA modifications at single-nucleotide resolution using rhodamine sequencing (Rho-seq). STAR Protoc. *3*, 101369.
25. Tang, Q., Nie, F., Kang, J., and Chen, W. (2021). mRNALocater: enhance the prediction accuracy of eukaryotic mRNA subcellular localization by using model fusion strategy. Mol. Ther. *29*, 2617–2623.
26. He, W., Jia, C., and Zou, Q. (2019). 4mCPred: machine learning methods for DNA N4-methylcytosine sites prediction. Bioinformatics *35*, 593–601.
27. Chen, K., Wei, Z., Zhang, Q., Wu, X., Rong, R., Lu, Z., Su, J., de Magalhães, J.P., Rigden, D.J., and Meng, J. (2019). WHISTLE: a high-accuracy map of the human N6-methyladenosine (m6A) epitranscriptome predicted using a machine learning approach. Nucleic Acids Res. *47*, e41.
28. Liu, Q., and Gregory, R.I. (2019). RNAmod: an integrated system for the annotation of mRNA modifications. Nucleic Acids Res. *47*, W548–W555.
29. Lv, H., Zhang, Z.-M., Li, S.-H., Tan, J.-X., Chen, W., and Lin, H. (2020). Evaluation of different computational methods on 5-methylcytosine sites identification. Brief. Bioinform. *21*, 982–995.
30. Zhou, Y., Zeng, P., Li, Y.-H., Zhang, Z., and Cui, Q. (2016). SRAMP: prediction of mammalian N6-methyladenosine (m6A) sites based on sequence-derived features. Nucleic Acids Res. *44*, e91.

31. Liu, K., and Chen, W. (2020). iMRM: a platform for simultaneously identifying multiple kinds of RNA modifications. Bioinformatics *36*, 3336–3342.
32. Zhu, H., Ao, C.-Y., Ding, Y.-J., Hao, H.-X., and Yu, L. (2022). Identification of D Modification sites using a random forest model based on nucleotide chemical properties. Int. J. Mol. Sci. *23*, 3044.
33. Dou, L., Zhou, W., Zhang, L., Xu, L., and Han, K. (2021). Accurate identification of RNA D modification using multiple features. RNA Biol. *18*, 2236–2246.
34. Xu, Z.-C., Feng, P.-M., Yang, H., Qiu, W.-R., Chen, W., and Lin, H. (2019). iRNAD: a computational tool for identifying D modification sites in RNA sequence. Bioinformatics *35*, 4922–4929.
35. Feng, P., Xu, Z., Yang, H., Lv, H., Ding, H., and Liu, L. (2019). Identification of D Modification sites by integrating heterogeneous features in Saccharomyces cerevisiae. Molecules *24*, 380.
36. Chen, W., Tang, H., Ye, J., Lin, H., and Chou, K.C. (2016). iRNA-PseU: identifying RNA pseudouridine sites. Mol. Ther. Nucleic Acids *5*, e332.
37. Song, B., Chen, K., Tang, Y., Wei, Z., Su, J., de Magalhães, J.P., Rigden, D.J., and Meng, J. (2021). ConsRM: collection and large-scale prediction of the evolutionarily conserved RNA methylation sites, with implications for the functional epitranscriptome. Brief. Bioinform. *22*, bbab088.
38. Wagih, O. (2017). ggseqlogo: a versatile R package for drawing sequence logos. Bioinformatics *33*, 3645–3647.
39. Vacic, V., Iakoucheva, L.M., and Radivojac, P. (2006). Two Sample Logo: a graphical representation of the differences between two sets of sequence alignments. Bioinformatics *22*, 1536–1537.
40. Dyubankova, N., Sochacka, E., Kraszewska, K., Nawrot, B., Herdewijn, P., and Lescrinier, E. (2015). Contribution of dihydrouridine in folding of the D-arm in tRNA. Org. Biomol. Chem. *13*, 4960–4966.
41. Väre, V.Y.P., Eruysal, E.R., Narendran, A., Sarachan, K.L., and Agris, P.F. (2017). Chemical and conformational diversity of modified nucleosides affects tRNA structure and function. Biomolecules *7*, E29.
42. Finet, O., Yague-Sanz, C., Krüger, L.K., Tran, P., Migeot, V., Louski, M., Nevers, A., Rougemaille, M., Sun, J., Ernst, F.G.M., et al. (2022). Transcription-wide mapping of dihydrouridine reveals that mRNA dihydrouridylation is required for meiotic chromosome segregation. Mol. Cell *82*, 404–419.e9.
43. Yang, H., Lv, H., Ding, H., Chen, W., and Lin, H. (2018). iRNA-2OM: a sequence-based predictor for identifying 2′-O-methylation sites in Homo sapiens. J. Comput. Biol. *25*, 1266–1277.
44. Chen, W., Feng, P., Tang, H., Ding, H., and Lin, H. (2016). RAMPred: identifying the N1-methyladenosine sites in eukaryotic transcriptomes. Sci. Rep. *6*, 31080.
45. Chen, W., Tang, H., and Lin, H. (2017). MethyRNA: a web server for identification of N6-methyladenosine sites. J. Biomol. Struct. Dyn. *35*, 683–687.
46. Chen, W., Yang, H., Feng, P., Ding, H., and Lin, H. (2017). iDNA4mC: identifying DNA N4-methylcytosine sites based on nucleotide chemical properties. Bioinformatics *33*, 3518–3523.
47. Nair, A.S., and Sreenadhan, S.P. (2006). A coding measure scheme employing electron-ion interaction pseudopotential (EIIP). Bioinformation *1*, 197–202.
48. Dou, L., Li, X., Ding, H., Xu, L., and Xiang, H. (2020). Prediction of m5C modifications in RNA sequences by combining multiple sequence features. Mol. Ther. Nucleic Acids *21*, 332–342.
49. Han, S., Liang, Y., Ma, Q., Xu, Y., Zhang, Y., Du, W., Wang, C., and Li, Y. (2019). LncFinder: an integrated platform for long non-coding RNA identification utilizing sequence intrinsic composition, structural information and physicochemical property. Brief. Bioinform. *20*, 2009–2027.
50. Bonidia, R.P., Sampaio, L.D.H., Domingues, D.S., Paschoal, A.R., Lopes, F.M., de Carvalho, A.C.P.L.F., and Sanches, D.S. (2021). Feature extraction approaches for biological sequences: a comparative study of mathematical features. Brief. Bioinform. *22*, bbab011.
51. Luong, T., Pham, H., and Manning, C.D. (2015). Effective approaches to attention-based neural machine translation. In Proceedings of the 2015 Conference on Empirical Methods in Natural Language Processing.
52. Zheng, G., Mukherjee, S., Dong, X.L., and Li, F. (2018). OpenTag: open attribute value extraction from product profiles. In Proceedings of the 24th ACM SIGKDD International Conference on Knowledge Discovery & Data Mining.
53. Vaswani, A., Shazeer, N., Parmar, N., Uszkoreit, J., Jones, L., Gomez, A.N., Kaiser, Ł., and Polosukhin, I. (2017). Attention is all you need. In Proceedings of the 31st International Conference on Neural Information Processing Systems.
54. Cordonnier, J.-B., Loukas, A., and Jaggi, M. (2019). On the relationship between self-attention and convolutional layers. In International Conference on Learning Representations (ICLR).
55. Kingma, D., and Ba, J. (2014). Adam: a method for stochastic optimization. In International Conference on Learning Representations (ICLR).

OMTN, Volume *31*

Supplemental information

Self-attention enabled deep learning of dihydrouridine (D) modification on mRNAs unveiled a distinct sequence signature from tRNAs

Yue Wang, Xuan Wang, Xiaodong Cui, Jia Meng, and Rong Rong

SUPPLEMENTAL TABLES

Table S1. Model performance of different deep learning methods for predicting dihydrouridine on mRNA.

**Modes**	**Methods**	**Sn** (%)	**Sp** (%)	**ACC** (%)	**F1**	**MCC**	**AUROC**
Training	Transformer	69.67	72.25	70.83	0.6986	0.3908	0.7362
	Local self-attention	80.55	77.78	79.17	0.7946	0.5895	0.8194
	CNN of one layer	90.74	85.19	87.96	0.8831	0.7608	0.8796
	CNN of two layers	90.02	86.67	88.33	0.8852	0.7674	0.8833
	ResNet	70.02	75.56	72.78	0.7158	0.4655	0.7873
	DPred	**92.59**	**87.78**	**90.27**	**0.8947**	**0.7826**	**0.9116**
Testing	Transformer	77.78	66.67	72.22	0.7368	0.4208	0.7654
	Local self-attention	83.33	72.22	77.78	0.7895	0.5590	0.8204
	CNN of one layer	88.89	77.78	86.11	0.8718	0.7324	0.8890
	CNN of two layers	94.44	83.33	88.89	0.8947	0.7826	**0.9035**
	ResNet	77.78	72.22	75.00	0.7568	0.5205	0.7870
	DPred	**94.44**	**88.89**	**91.66**	**0.9189**	**0.8062**	0.9027

Notes: DPred was compared with five other deep learning approaches, including local self-attention, CNNs, ResNet (residual connection + CNN) and Transformer (multi-heads self-attention). The results indicated that convolutional neural networks play crucial roles in site prediction. DPred, a combination of CNNs and local self-attention outperformed other methods that only used either one of them individually, and achieved the best performance in our study.

Table S2. DPred model performance on dihydrouridine tRNA datasets in different species.

Species	Sn (%)	Sp (%)	ACC (%)	F1 Scores	MCC	AUROC
S.cerevisiae	94.87	93.59	94.23	0.9427	0.8847	0.9675
H.sapiens	86.36	95.45	92.86	0.9315	0.8603	0.9731
E.coli	97.14	91.43	94.29	0.9444	0.8872	0.9857
M.musculus	92.43	86.77	91.67	0.9289	0.8346	0.9290

Table S3. DPred model performance under mature RNA / full transcript datasets.

Datasets	Sn (%)	Sp (%)	ACC (%)	F1 Scores	MCC	AUROC
Full transcript (101nt)	63.15	84.21	73.68	0.7059	0.4845	0.8333
mRNA (101 nt)	83.33	77.78	80.56	0.8108	0.6121	0.8642
Full transcript (41 nt)	78.94	89.47	84.21	0.8333	0.6880	0.8823
mRNA (41 nt)	94.44	88.89	91.66	0.9189	0.8062	0.9027

Table S4. EIIP value for each nucleotide.

Nucleotide	EIIP
A	0.1260
U	0.1335
G	0.0806
C	0.1340

Table S5. The proposed DPred architecture.

Layers	Output size
Input	(-, 41, 4)
Self_attention	(-, 41, 4)
Conv	(-, 41, 4, 100)
Max_pooling	(-, 20, 2, 100)
Flatten	(-, 4000)
Dropout	(-, 4000)
Dense	(-, 100)
Output	(-, 1)

SUPPLEMENTAL FIGURES

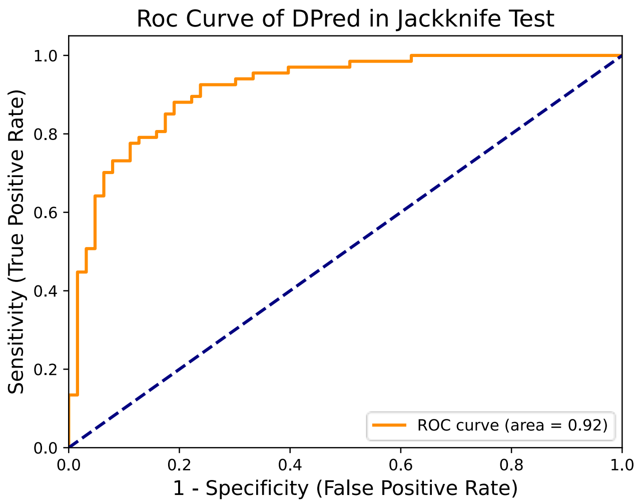

Figure S1. ROC curve on training data under jackknife cross-validation. The area under the receiver operating characteristic curve is 0.92.

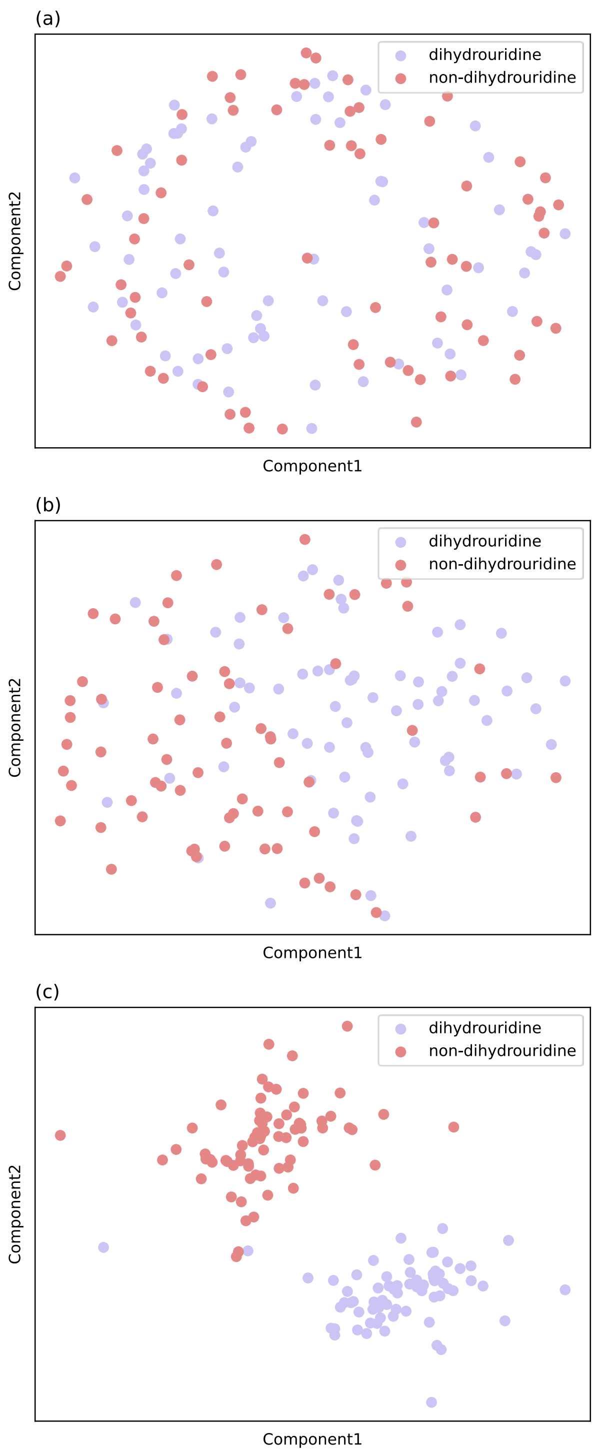

Figure S2. t-SNE plots of different layers in DPred. Feature visualization of (a) original input sequences, (b) flatten layer and (c) the last dense layer from the well-trained model.

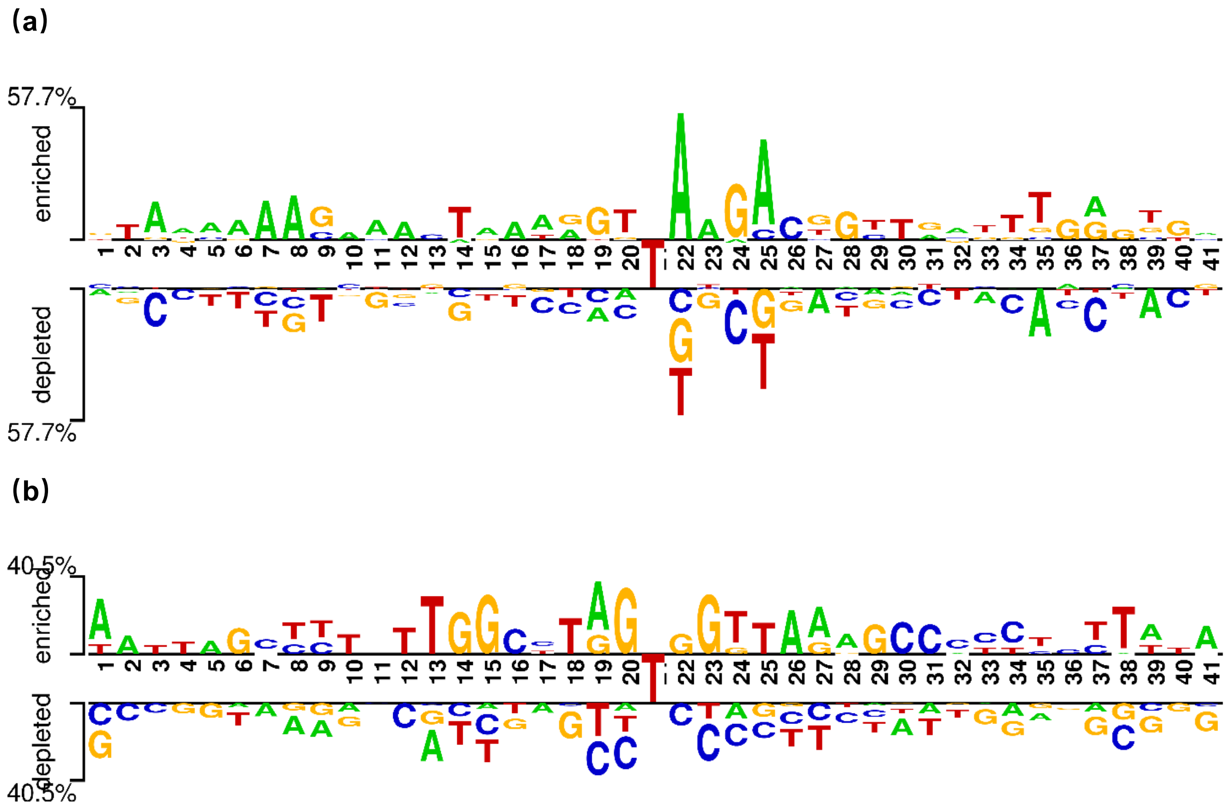

Figure S3. Two Sample Logo of the differences between positive and negative sequences. Demonstration of nucleotide composition preferences between dihydrouridine and non-dihydrouridine for mRNA (a) and tRNA (b) datasets.

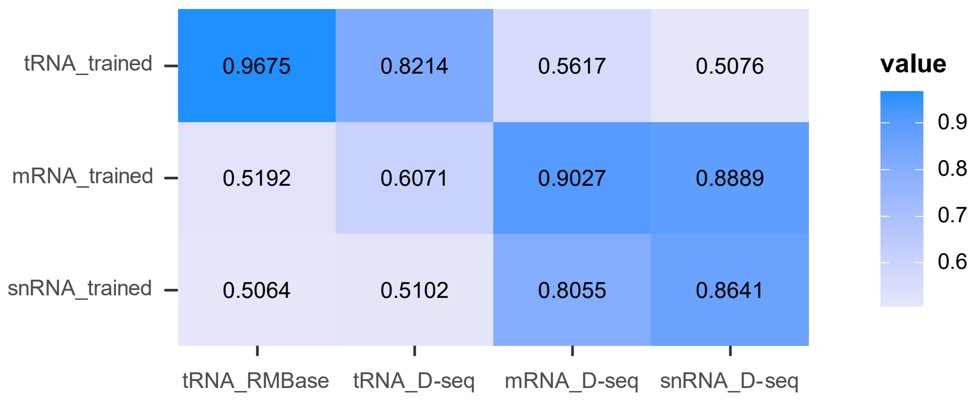

Figure S4. Heat map shows cross RNA types prediction accuracies. We trained DPred on tRNA, mRNA and snRNA training datasets independently, and made prediction on testing datasets of their own and other RNA types.
